# Whole Genome Shotgun Sequencing Shows Selection on *Leptospira* Regulatory Proteins during in vitro Culture Attenuation

**DOI:** 10.4269/ajtmh.15-0401

**Published:** 2016-02-03

**Authors:** Jason S. Lehmann, Victoria C. Corey, Jessica N. Ricaldi, Joseph M. Vinetz, Elizabeth A. Winzeler, Michael A. Matthias

**Affiliations:** Division of Infectious Diseases, School of Medicine, University of California, La Jolla, San Diego, California; Biomedical Sciences Graduate Program, University of California, La Jolla, San Diego, California; Department of Pediatrics, School of Medicine, University of California, La Jolla, San Diego, California; Instituto de Medicine Tropical “Alexander von Humboldt,” Department of Cellular and Molecular Sciences, Faculty of Sciences and Laboratory of Research and Development, Universidad Peruana Cayetano Heredia, Lima, Peru

## Abstract

Leptospirosis is the most common zoonotic disease worldwide with an estimated 500,000 severe cases reported annually, and case fatality rates of 12–25%, due primarily to acute kidney and lung injuries. Despite its prevalence, the molecular mechanisms underlying leptospirosis pathogenesis remain poorly understood. To identify virulence-related genes in *Leptospira interrogans*, we delineated cumulative genome changes that occurred during serial in vitro passage of a highly virulent strain of *L*. *interrogans* serovar Lai into a nearly avirulent isogenic derivative. Comparison of protein coding and computationally predicted noncoding RNA (ncRNA) genes between these two polyclonal strains identified 15 nonsynonymous single nucleotide variant (nsSNV) alleles that increased in frequency and 19 that decreased, whereas no changes in allelic frequency were observed among the ncRNA genes. Some of the nsSNV alleles were in six genes shown previously to be transcriptionally upregulated during exposure to in vivo-like conditions. Five of these nsSNVs were in evolutionarily conserved positions in genes related to signal transduction and metabolism. Frequency changes of minor nsSNV alleles identified in this study likely contributed to the loss of virulence during serial in vitro culture. The identification of new virulence-associated genes should spur additional experimental inquiry into their potential role in *Leptospira* pathogenesis.

## Introduction

Leptospirosis, caused by pathogenic species of the genus *Leptospira,* is an emerging zoonotic infection of global distribution.^1^ Recent estimates by the Leptospirosis Burden Epidemiology Reference Group have placed the number of hospitalized cases at over 500,000 per year^2^; this, more than likely, is an underestimate of the true burden of disease due primarily to inadequate diagnostics, a lack of clinical awareness, and poor surveillance.^3^ Transmission to humans occurs via exposure to contaminated water and wet soil or infected tissues and urine from chronically colonized reservoir hosts. Humans living in poverty with poor sanitation are at greatest risk, particularly during seasonal flooding, monsoons, and tropical cyclones.^1^,^3^

**Figure 1.**
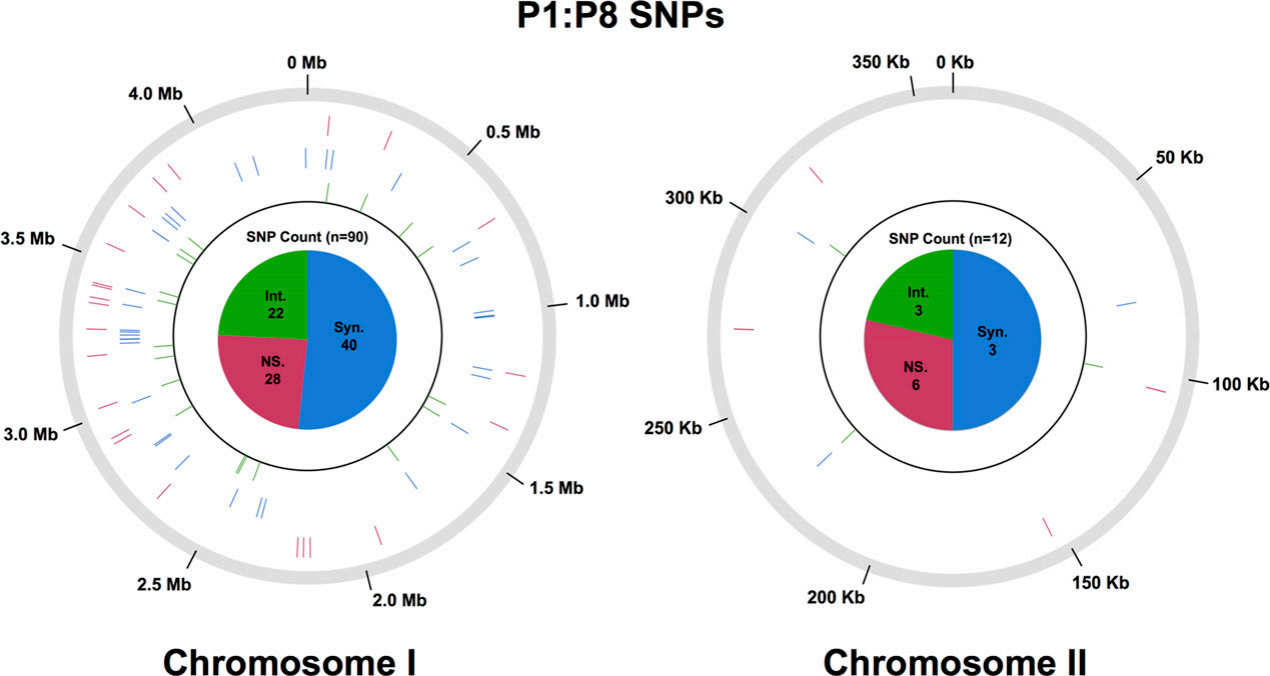
Genomic locations of single nucleotide variants (SNVs) that change allelic frequency from P1 to P8A. Genomic location of study identified SNVs in the reference genome of *Leptospira interrogans* serovar Lai strain 56601 that significantly changed in allelic proportionality during culture attenuation from a virulent P1 strain to an attenuated P8A strain. Individual hash marks represent the genomic location of genes containing SNVs, and are color coded in concentric circles. Red = nonsynonymous; blue = synonymous; green = intergenic. The total number of genes containing each type of SNV is represented by the pie chart in the center of each of the chromosome representations.

The *Leptospira* genus includes at least 22 species classified into three large subgroups based on 16S rDNA phylogeny, in vitro growth characteristics, and virulence.^4^–^7^ There are 15 recognized pathogenic species. Group I pathogens (Figure 1

) comprise > 250 serotypes and cause disease varying in severity, ranging from subclinical infections to severe disease—often associated with renal failure and pulmonary hemorrhage—and death.^8^ By contrast, group II species grow better in culture and cause predominantly mild, self-resolving illness without fatal complications. Of the pathogenic species, *Leptospira interrogans*, the leading cause of leptospirosis-associated morbidity and mortality in humans, is the most extensively studied species.^1^,^2^ Nonetheless, the molecular mechanisms underlying *L. interrogans* pathogenesis remain largely unknown primarily because targeted gene knockouts in pathogenic *Leptospira* is inefficient and technically challenging.^9^ Despite this barrier to progress in the field, transposon mutagenesis, first reported by Bourhy and others^10^ and Murray and others,^11^ has been successful. Though technically difficult, targeted gene knockouts have also been described and used to validate a handful of *Leptospira* virulence-related genes (e.g., *fliY*, *colA*, *mce*).^12^–^14^

Given the difficulty of targeted gene knockouts, systems-based approaches, including transcriptome and comparative genome analysis, have been used to identify potential virulence candidates. Microarrays have been applied to investigate the transcriptional response of pathogenic *Leptospira* to various “host-like” conditions including temperature,^15^,^16^ serum,^17^ physiological osmolarity,^18^ iron depletion,^19^ and host immune cells.^20^ Recent RNA-seq experiments have further improved our understanding of global transcriptional responses during *Leptospira* growth in vivo.^21^,^22^ In addition, our group has applied comparative genome analysis to identify 452 conserved pathogen-specific genes that likely play a role in *Leptospira* pathogenesis.^4^,^23^–^27^ Nonetheless, the contribution of individual genes or combinations of genes to the overall virulence phenotype of pathogenic *Leptospira* remains poorly understood.

In a previous independent study, we used reference-guided assemblies to identify inactivating nonsynonymous single nucleotide variant (nsSNVs) in 11 putative virulence-associated genes that had emerged after passaging a P1 isolate for 18 subcultures including a family of virulence-modifying proteins upregulated during in vivo in an acute hamster infection model.^28^ However, in this experiment, we considered only dominant alleles in P1 and P18 isolates. Here, in an independent attenuation experiment, we serially in vitro passaged the P1 isolate (LD_50_ < 100 *Leptospira*) into an avirulent derivative (P8A, LD_50_ > 10^8^). We define the cumulative genome changes accompanying this observed loss of virulence by comparing the genomes of the parental strain and its isogenic, attenuated derivative through the use of next-generation sequencing and a custom SNV calling pipeline.^29^

## Methods

### Ethics statement.

The experimental animal work was carried out in accordance with the recommendations in the Guide for the Care and Use of Laboratory Animals of the National Institutes of Health in Association for Assessment and Accreditation of Laboratory Animal Care (AAALAC)-approved facilities, and was approved by the Institutional Animal Care and Use Committee of the University of California, San Diego under protocol S03128H.

### Attenuation of *L. interrogans* serovar Lai strain 56601.

#### Generation of the P1 isolate of L. interrogans serovar Lai strain 56601.

*Leptospira interrogans* serovar Lai strain 56601 was kindly provided by David Haake (University of California Los Angeles, Los Angeles, CA), and was passaged through 3-week-old male Golden Syrian hamsters (*N* = 3, Charles Rivers Laboratories, Hollister, CA) to ensure a virulent phenotype. The initial three hamsters were each injected intraperitoneally (IP) with approximately 10^7^ *Leptospira* in 1 mL of Ellinghausen-McCullough-Johnson-Harris *Leptospira* culture media (EMJH; BD Difco, Franklin Lakes, NJ). Four days post inoculation the animals were killed, the livers were harvested, macerated with a sterile scalpel blade, pooled in 5-mL sterile phosphate-buffered saline, then made into a slurry by repeatedly passing through a 22-gauge needle; 1 mL of this homogenate was then used to inject each of a second group (*N* = 3) of hamsters IP. The liver homogenization procedure was repeated 4 days later, and a third group (*N* = 3) of hamsters were injected, also IP. Four days after the IP injection of liver homogenate into the third group, the animals were killed, and livers harvested aseptically. Approximately 10 mg of minced liver tissue was then used to inoculate EMJH semisolid medium supplemented with 5-fl.^30^ The semisolid culture was incubated at 25°C and monitored for *Leptospira* growth by dark field microscopy. Once growth occurred, 100 μL of this culture was used to inoculate 20 mL of sterile EMJH media, and the culture was incubated at 28°C on a rotary shaker, and was designated P1.

#### Genomic DNA isolation of P1 isolate.

Approximately 10^7^ *Leptospira* from 1 mL of EMJH P1 culture were spun down in a microcentrifuge (10,000 rpm, 5 minutes). Genomic DNA was then isolated from the cell pellet using the DNEasy Blood and Tissue kit (Qiagen, Valencia, CA) according to manufacturer's instructions. Eluted DNA was stored at −20°C for later sequencing.

#### LD_50_ determination of P1 isolate.

*Leptospira* cells were counted using a Petroff-Hauser counting chamber (Hausser Scientific, Horsham, PA) under dark field microscopy. Challenge doses of 10^2^, 10^3^, 10^4^, 10^5^, 10^6^, 10^7^, and 10^8^ *Leptospira*/mL in sterile EMJH were then prepared based on observed counts. For each dilution group, 3-week-old male Golden Syrian hamsters (*N* = 3, Charles Rivers Laboratories) were each injected IP with 1 mL of the appropriate challenge dose. Animals were monitored for 21 days and euthanized when moribund. The LD_50_ was defined as the last dose in which two-thirds of the animals died after challenge.

#### In vitro EMJH culture-passage attenuation of the virulent P1 isolate into P8 isolate.

The P1 isolate EMJH culture was subcultured by transferring 2 mL into 18 mL of sterile EMJH media (thus becoming P2A), and incubated at 28°C on a rotary shaker for 14 days. This process was repeated iteratively for a total of seven subcultures, with the final subculture being designated P8A (∼400 generations from the P1 parent culture). Genomic DNA extraction and LD_50_ determination were then performed exactly as described for the P1 isolate.

### Genomic library preparation and assembly.

Genomic DNA libraries were normalized to 0.2 ng/μL and prepared for sequencing using the Illumina Nextera XT Kit (Illumina, San Diego, CA) whole genome resequencing library according to manufacturer's instructions, using the Illumina protocol of tagmentation followed by ligation (v. 2013; Illumina, Inc., San Diego). DNA libraries were clustered and run on an Illumina HiSeq 2500 platform (Illumina) with PE100 on Rapid Run mode. Base calls were made using CASAVA v 1.8+ (Illumina).

Sequences were processed though the PLATYPUS pipeline (Winzeler Lab, UCSD, San Diego, CA).^29^ In brief, reads were aligned to the reference *L. interrogans* serovar Lai strain 56601 genome (NC_004342 and NC_004343) using Burrows-Wheeler Aligner,^31^ and unmapped reads were filtered using SAMtools.^32^ SNVs were then initially called using Genome Analysis Toolkit^33^,^34^ and filtered using default filter values in PLATYPUS. Although the filters were initially designed for *Plasmodium falciparum*, they resulted in high sensitivity (93.4%) and specificity (91.2%) for *L. interrogans* as well when screening for known SNVs between the 56601 and IPAV *L. interrogans* serovar Lai strains. After alignment, read depth per nucleotide identity at every position was called using SAMtools *mpileup*, which were then converted into proportional nucleotide identities per base. These proportions were then compared using a custom script testing for multi-comparison significant changes in allelic proportion across the entire genome. For two proportions *x*_1_/*n*_1_ and *x*_2_/*n*_2_ reads, our comparison statistic was

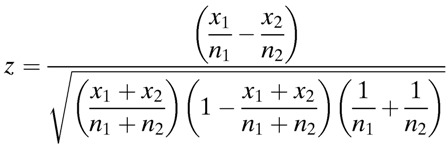


This statistic is an expansion of the simple two-proportional *z*-test for differences between two populations. This assumption is reasonable as each read serves as an independent random test of the nucleotide identity of the population, though significant error terms do exist. This number was then converted to a p-statistic using the total read depth and corrected using the Bonferroni method, as assumption about the independence of allelic frequency at multiple polymorphic sites may not hold. A list of sites that underwent statistically significant changes were then exported and annotated using a custom script.

### Clusters of orthologous groups' functional category analysis of nsSNV-containing genes.

Genes identified as containing nsSNVs with increasing allele frequencies in P8 were assigned to clusters of orthologous groups (COG) categories using the National Center for Biotechnology Information> conserved domain webpage (http://www.ncbi.nlm.nih.gov/Structure/cdd/cdd.shtml), and compared with the genome-wide predicted COG frequencies for *L. interrogans* serovar Lai strain 56601 obtained from the spirochete genome browser webpage (http://sgb.fli-leibniz.de/cgi/index.pl). Statistical significance was assessed via χ^2^ analysis using Fisher's exact test with a Bonferroni correction to account for multiple comparisons in Graphpad Prism (GraphPad Software, Inc., La Jolla, CA).

### Pan genus comparative genome analysis of study-identified genes.

The following genomes, consisting of a representative grouping all 20 *Leptospira* species, were used to analyze the presence of homologs of study-identified genes in other *Leptospira* species:

*Leptospira alexanderi* sv. Manhoa 3 str. L 60^T^ (Genbank: AHMT00000000), *Leptospira alstoni* sv. Pingchang str. 80-412 (Genbank: AOHD00000000), *Leptospira biflexa* sv. Patoc str. Patoc I Paris (Genbank: CP000786), *Leptospira borgpetersenii* sv. Javanica str. UI 09931 (Genbank: AHNP00000000), *Leptospira broomii* sv. Hurstbridge str. 5399^T^ (Genbank: AHMO00000000), *Leptospira fainei* sv. Hurstbridge str. BUT 6^T^ (Genbank: AKWZ00000000), *Leptospira inadai* sv. Lyme str. 10^T^ (Genbank: AHMM00000000), *L. interrogans* sv. Copenhageni str. Fiocruz L1-130 (Genbank: AE016823), *L. interrogans* sv. Lai str. 56601 (Genbank: AE010300), *Leptospira kirschneri* sv. Cynopteri str. 3522 C^T^ (Genbank: AHMN00000000), *Leptospira kmetyi* sv. Malaysia str. Bejo-Iso9^T^ (Genbank: AHMP00000000), *Leptospira licerasiae* sv. Varillal str. VAR 010^T^ (Genbank: AHOO00000000), *Leptospira meyeri* sv. Hardjo str. Went 5 (Genbank: AKXE00000000), *Leptospira noguchii* sv. Panama str. CZ 214^T^ (Genbank: AKWY00000000), *Leptospira santarosai* sv. Shermani str. 1342K^T^ (AOHB00000000), *Leptospira terpstrae* sv. Hualin str. LT 11-33^T^ (Genbank: AOGW00000000), *Leptospira vanthielii* sv. Holland str. WaZ Holland (Genbank: AOGY00000000), *Leptospira weilii* sv. undetermined str. LNT 1234 (Genbank: AOHC00000000), *Leptospira wolbachii* sv. Codice str. CDC (Genbank: AOGZ00000000), *Leptospira wolffii* sv. undetermined str. Khorat-H2^T^ (Genbank: AKWX00000000), *Leptospira yanagawae* sv. Saopaulo str. Sao Paulo^T^ (Genbank: AOGX00000000).

Genes were considered homologs if they were bidirectional best hits^35^,^36^ using Basic Local Alignment Search Tool (BLAST) with cutoff values of 70% query coverage, e-values < 1e^−3^, and 30% identity.

### Amino acid residue conservation analysis of study-identified nsSNV positions.

Domain architecture analysis was performed on the protein sequences for LA_2704 (NP_712885.1), LA_2930 (713110.1), LA_2950 (NP_713130.1), LA_3455 (NP_713635.1), LA_3725 (NP_713905.1), and LA_3834 (NP_714014.1) using Simple Modular Architecture Research Tool (SMART)^37^ and protein structure prediction server (PSIPRED),^38^,^39^ and represented graphically at http://prosite.expasy.org/mydomains.

Multiple sequence alignments (MSAs) of the homologs (defined by 70% query coverage, e-values < 1e^−3^, and 30% identity BLAST cutoffs) of each of these six genes were constructed by aligning sequences obtained from the Pathosystems Resource Integration Center database (http://patricbrc.org) using the CLUSTAL X alignment program freely available at http://www.clustal.org/clustal2/#Download. The accession numbers used in the alignments (Supplemental Table 1) are a representative collection of homolog sequences from each of the 20 species in the *Leptospira* genus in which homolog sequences could be identified. The LA_2704 alignment contained 40 homolog sequences, LA_2930 had 26 homologs, LA_2950 had 41 homologs, LA_3455 had 41 homologs, LA_3725 had 19 homologs, and LA_3834 had 45 homologs.

MSAs were then used to predict protein residue conservation based on Jensen-Shannon Divergence (JSD).^40^ Conservation scores were then graphed using Microsoft Excel (Redmond, WA).

The proportion of sequencing reads from the P8A strain coding for the nsSNV amino acid was compared with the proportion of the same mutant residues in homolog MSAs from the entire pan-*Leptospira* genome using a Fisher's exact test for each of the six genes. Results were considered statistically significant at *P* < 0.05.

### Identification of potential ncRNAs in the *L. interrogans* serovar Lai strain 56601 genome.

To identify novel ncRNA loci within the *L. interrogans* Lai strain 56601 genome, we first aligned the *L. interrogans* Lai 56601 (Genbank: AE010300), *L. kirschneri* Cynopteri 3522 C (Genbank: AHMN00000000), and *L. noguchii* Panama CZ214^T^ (Genbank: AKWY00000000) genomes using the progressive Cactus algorithm.^41^,^42^ The whole genome alignment was then used as input for RNAz (with default settings: -w 120 and -s 120) for prediction of structural RNAs^43^ and then putative ncRNA loci identified and annotated using the nocoRNAc pipeline.^44^ Predicted loci that could not be annotated using the Rfam database were considered potentially novel ncRNA genes.

## Results

### Culture passage-based attenuation of *L. interrogans* serovar Lai strain 56601.

The P1 isolate was derived from *L. interrogans* serovar Lai strain 56601 that had been serially passaged 3X in vivo to ensure a virulent phenotype.^28^ The LD_50_ was determined to be < 10^2^ *Leptospira*.^28^ The P1 isolate was serially passaged in vitro in liquid *Leptospira* culture medium for ∼400 generations (16 weeks) to become P8A. The LD_50_ of the P8A isolate was determined to be > 10^8^ *Leptospira*, administered IP, indicating a complete loss of virulence. After in vitro passage, genomic DNA was isolated from both the P1 and P8A strains and frozen before sequencing.

### Identification of SNV alleles differing in frequency between the attenuated and parental strains.

Cumulative changes occurring during adaptation to in vitro growth, and associated with loss of virulence, were studied at the whole genome level. Genomic DNA from the nonclonal parental strain, P1, and from the attenuated isogenic derivative, P8A, was sequenced on an Illumina platform using paired-end 100-bp reads to a mean coverage of greater than 250X. For strain P1, 15,492,436 reads were generated covering 99.4% of the *L. interrogans* serovar Lai reference genome (4.689 Mb), and 15,651,273 reads were generated from P8A covering 99.9% of the reference genome (Table 1). In addition, > 99% of the reads from both the P1 and P8A samples aligned to the *L. interrogans* Lai strain 56601 genome, indicating high sample purity.

Variants were called and compared using a modified automated PLATYPUS genome analysis pipeline.^29^ PLATYPUS aligned reads from each sequencing run (P1 and P8A) to the reference Lai genome^27^ and identified SNVs using a default list of filters for each set of sequencing files. Given that the bacterial populations were not clonal, an allele frequency was calculated at each polymorphic site using the number of aligned reads metric for the P1 and P8A isolate (Supplemental Table 1). From this analysis, 99 SNVs were identified as having undergone a significant change in allele frequency between P1 and P8A, as determined by a two-proportional *z*-test before Bonferroni correction. Alternate nucleotides in these positions would result in 43 SNVs encoding synonymous amino acid substitutions, 34 encoding nonsynonymous amino acid substitutions, and 25 intergenic SNVs (Figure 1, see Supplemental Table 1 for complete listing of variants). In the P8A genome, all of these minor alleles had changed allele frequencies by at least 5% compared with the P1 genome and vice versa.

### Analysis of nsSNVs with allelic frequencies that increased during attenuation.

**Figure 2.**
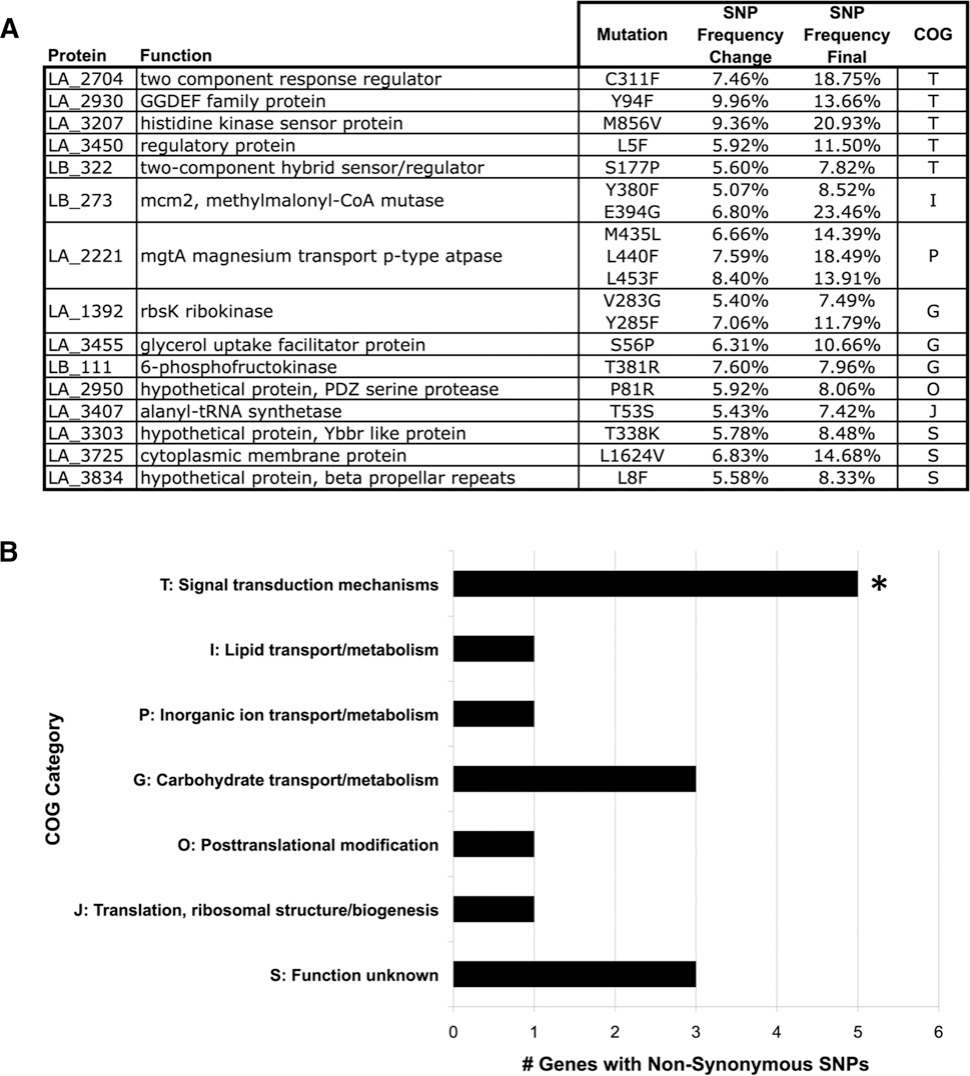
Clusters of orthologous group analysis of genes containing nonsynonymous single nucleotide variants (nsSNVs) that increased allelic frequency from P1 to P8A. The nsSNVs that increased allelic frequency during the attenuation of the virulent P1 strain of *L. interrogans* serovar Lai strain 56601 into the avirulent isogenic P8A strain are listed in (**A**). The nsSNV-containing genes in each group were then organized by clusters of orthologous groups (COG) category.^45^ Asterisks denote an enrichment of a particular COG category compared with genome-wide expected percentages of genes in each category (Fisher's exact test, *P* value given in figure) in (**B**). Total number of genes in *Leptospira interrogans* serovar Lai strain 56601 genome in represented COG categories: T = 233, I = 110, *P* = 134, G = 131, O = 118, J = 172, S = 853. Total number of predicted genes = 3,683.

Since amino acid coding changes can alter overall functionality of the gene in which they reside, and may contribute to the observed loss of virulence in the P8A strain, we further examined the nsSNVs that were identified during our genomic comparisons. There were 15 genes that contained nsSNVs that increased in frequency during the course of the attenuation (Figure 2A

). To determine if the genes containing these nsSNVs were biased toward any particular biological function, they were organized by COG category,^45^ and the observed proportions were compared with their genome-wide expected frequencies. This approach identified a strong enrichment for genes involved in signal transduction mechanisms (Figure 2B). Of the 3,683 genes in the genome, 233 are annotated as involved in signal transduction and comprised five of the 15 in our set (*P* = 0.01). We also noted that three genes, *rbsK, mgtA*, and *mcm2* (encoding a putative ribokinase, a magnesium transporter, and methylmalonyl-CoA mutase, respectively), contained multiple SNVs. This is a higher number than that would be expected due to chance alone, and because these genes all have functions related to core metabolic pathways and cofactor biosynthesis, their allele frequency increase may be a result of bacterial adaptation to long-term in vitro culture conditions.

To infer additional possible biological significance of these genes, we performed a meta-analysis of previously published data showing transcriptional responses of *L. interrogans* under several surrogate in vivo conditions including temperature, physiological osmolarity, iron depletion, exposure to host innate immune cells, and peritoneal culture of pathogenic *Leptospira* in dialysis membrane chambers.^15^,^16^,^18^–^21^ Of the 15 genes identified by this study as harboring nsSNVs of increasing allele frequency, six (LA_2704, LA_2930, LA_2950, LA_3455, LA_3725, and LA_3834) were previously reported to be upregulated in at least one set of in vitro surrogate experimental conditions.

**Figure 3.**
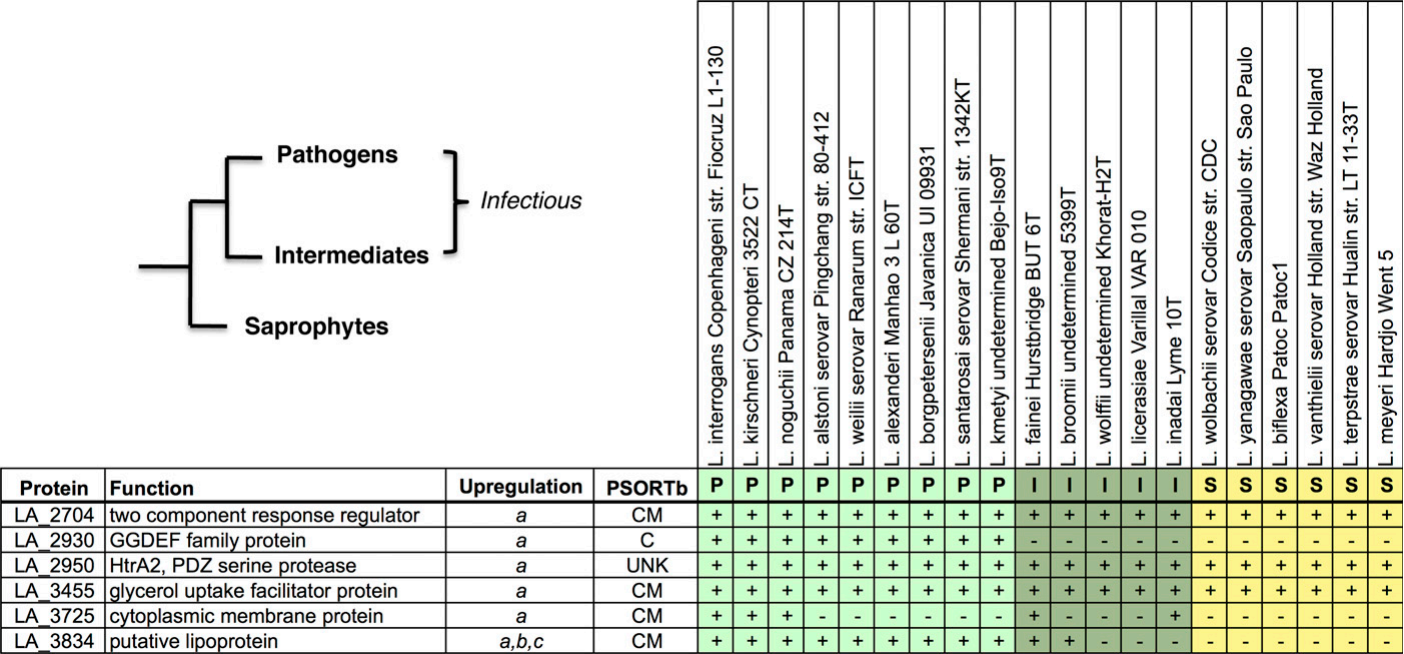
Homolog identification and characterization of potential virulence-associated genes in other *Leptospira* species. Potential virulence-associated genes identified in this study include LA_2704 (NP_712885.1), LA_2930 (NP_713110.1), LA_2950 (NP_713130.1), LA_3455 (NP_713635.1), LA_3725 (NP_713905.1), and LA_3834 (NP_714014.1). Previous studies have shown these genes to be upregulated by *Leptospira interrogans* during exposure to host-like conditions (*a* = response to host innate immunity^20^; *b* = response to host physiological osmolarity,^18^ *c* = response to host cues during in vivo culture in intraperitoneal dialysis cassettes^21^). The PSORTb predicted subcellular locations of each of these proteins are listed. The presence of orthologous genes (defined as bidirectional best Basic Local Alignment Search Tool [BLAST] hits with minimum 70% query coverage, e-values < 1e^−3^, and 30% identity) was also determined for each of the 20 species in the *Leptospira* genus. P = pathogenic species; I = intermediate pathogens; S = saprophytic species). A schematic representation of these three clades of the genus was included for clarity.

To gain further insight into how these genes might contribute to the pathogenicity of *Leptospira* and their overall prevalence in the genus, the subcellular locations of the proteins they encode were predicted using PSORTb v. 3.0 (http://psort.org/psortb/index.html),^46^ and the prevalence of gene homologs across all 20 species of the *Leptospira* pan-genome was also determined. Genes in other *Leptospira* species were considered homologous to our study-identified genes if they were reciprocal best BLAST hits using filters of 70% query length, e-value < 1e^−3^, and 30% identity match (Figure 3

). This analysis revealed that five of the six genes (the subcellular location of LA_2950 could not be determined by the algorithm) were predicted to reside inside the bacterial cell, indicating that these proteins are likely not the ultimate effectors of *Leptospira* pathogenesis, like toxins or other secreted factors, but may contribute to upstream signaling processes or metabolic capability. The pan-genus conservation analysis showed that three genes (LA_2930, LA_3725, and LA_3834) are found only in infectious *Leptospira* species and may have particularly relevant pathogenesis-related functions.

### Pan-*Leptospira* genomic analysis of amino acid residue conservation at nsSNV positions in homologs of attenuation-identified genes.

We conducted a three-part analysis of six genes of interest (Figure 3) to determine if the nsSNVs in these genes caused amino acid changes in evolutionarily conserved residues. First, protein domain architecture was evaluated using SMART^37^ and PSIPRED.^38^,^39^ Next, we generated MSAs using homologous sequences from the 20 species pan-*Leptospira* genome for each of these genes. These MSAs were used to generate amino acid conservation scores for each residue in a respective gene based on JSD (scores above 0.8 are considered highly conserved, those less than 0.4 are considered disordered).^40^ Finally, we compared the proportion of sequencing reads from the P8A strain coding for the nsSNV amino acid to the proportion of the same mutant residue in homologs from the entire pan-*Leptospira* genome using a Fisher's exact test with the following results.

#### LA_2704.

**Figure 4.**
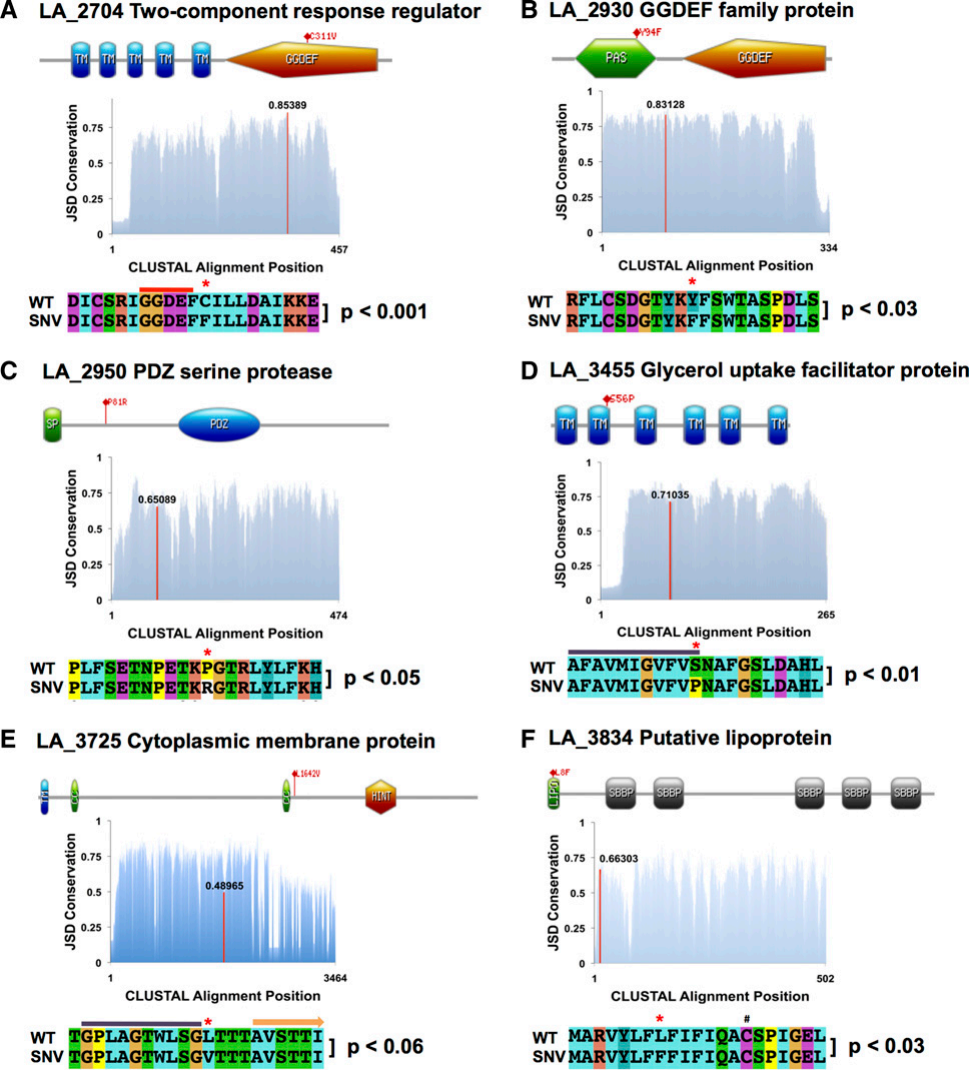
Amino acid conservation analysis at nonsynonymous single nucleotide variants (nsSNV) positions in study identified gene homologs across the *Leptospira* genus. Protein domain analysis was conducted for all genes and results are represented as diagrams at the top of panels **A**–**E** that include the nsSNV position for each gene (TM = transmembrane domain; PAS = Per-ARNT-Sim domain; GGDEF = diguanylate cyclases domain; SP = signal peptide; PDZ = PDZ serine protease domain; CC = coiled-coil domain; HINT = hedgehog intein domain; LIPO = *Leptospira* lipobox; SBBP = seven-bladed beta propeller domain). In addition, a Jensen-Shannon Divergence (JSD) estimate of amino acid residue conservation is represented graphically with the nsSNV residue highlighted as a red line with the conservation score indicated above it (scores above 0.8 indicate high conservation, those below 0.4 indicate disorder). Finally, at the bottom of each panel, an alignment schematic with the nsSNV position highlighted by a red asterisk is presented with the probability from a Fisher's exact test comparison of the number of P8A sequencing reads coding for the mutant amino acid to the genome-wide prevalence of that same residue in homologs of that particular gene. In (**A**), the red line above the alignment indicates the position of the catalytic residue of the protein. In (**D**), the purple line represents a conserved α helix. In (**E**), the purple line represents a predicted α helix, and the orange arrow represents a predicted beta-sheet secondary structure. In (**F**), the hash mark denotes the cysteine residue that is lipidated.

Diguanylate cyclases participate in the formation of the ubiquitous second messenger, cyclic diguanylate monophosphate (cyclic-di-GMP), involved in bacterial virulence, biofilm formation, and persistence.^47^,^48^ The nonsynonymous C311F substitution in this GGDEF, diguanylate cyclase is C-terminal to the catalytic core of this protein by one amino acid residue (Figure 4A

).^49^ The wild-type cysteine residue is conserved in every single homolog evaluated in this study, which is reflected by the high conservation score obtained from JSD analysis. The proportion of phenylalanine substitutions observed in the P8A strain represents a highly significant divergence from the genus-wide residue conservation at this position (*P* < 0.001).

#### LA_2930.

The Y94F substitution in this Per-ARNT-Sim-(PAS)-GGDEF predicted signaling protein falls within the PAS sensor domain (Figure 4B). PAS domains detect a large range of chemical and physical signals and then regulate the activity of their covalently linked effector domains, often by promoting the formation of dimers (a process required for proper GGDEF domain function). We could not deduce any insight into the particular ligands to which the PAS domain of this protein may bind, as the range of potential signals is diverse (ranging from oxygen tension to small metabolites and to light itself) and on average, the pairwise identity shared between PAS domains is less than 20%.^50^ Nonetheless, conservation analysis revealed that this position is highly conserved in *Leptospira* with significant divergence (*P* < 0.03) away from conservation status in the P8A attenuated strain.

#### LA_2950.

post synaptic density protein (PDZ) serine proteases are a unique family of proteins that form higher order oligmeric structures and have been demonstrated to degrade misfolded proteins in the periplasm of bacteria.^51^ The P81R nsSNV in this PDZ serine protease was found to occur in an in-silico predicted coil to sheet transition, indicating that the wild-type proline residue may serve a structural role (Figure 4C). JSD conservation analysis revealed the site to be moderately conserved within the *Leptospira* genus. The P8A arginine substitution at this residue was significant (*P* < 0.05), and was not observed in any of the protein homologs evaluated. Interestingly, domain architecture analysis revealed an N-terminal signal peptide indicating that this protein potentially has extracellular function.

#### LA_3455.

This protein is a transmembrane nonselective transport channel found in the inner membrane of gram-negative bacteria that facilitates the diffusion of glycerol.^52^ The S56P substitution in this protein was a significant divergence from genus-wide expected residues (*P* < 0.01) (Figure 4D). The conserved residue position lies at the end of one of the eight α-helical regions of the aquaglyceroporin. The tight spatial arrangement of these helices to one another is essential for the proper function of the protein's glycerol-conducting channel,^53^ and the proline substitution in the P8A population of *Leptospira* could conceivably introduce a structural change that would alter its transport efficiency.

#### LA_3725.

Domain analysis of the large LA_3725 protein revealed a single N-terminal transmembrane domain and a pre-toxin Hedgehog/Intein (HINT) domain (Pfam PF07591) nearer the C-terminal end of the coding region. The HINT superfamily belongs to a system of proteases that in bacteria are usually found N-terminal to a toxin module in polymorphic toxin systems,^54^,^55^ and are believed to release the toxin domain via autoproteolysis. The L1624V nsSNV position lies at the in silico predicted transition of an α helix to a coiled secondary structure in a region of low sequence conservation (Figure 4E). MSA analysis revealed that the P8A proportion of nsSNV reads was not statistically significant compared with genus-wide expected ratios.

#### LA_3834.

The nsSNV position identified in the attenuated P8 strain codes for an L8F substitution in the N-terminal lipobox^56^ of this protein (Figure 4F). This amino acid substitution occurs at a moderately conserved residue according to JSD analysis, that is, seven residues upstream of the cysteine residue that is lipidated during export through the bacterial inner membrane. Although there appeared to be some flexibility in the amino acid conservation at the SNV position, genus-wide analysis revealed that no homologs contained the mutant phenylalanine at this position, indicating a significant divergence from expected proportions (*P* < 0.03).

### Intergenic SNV analysis and novel ncRNA prediction.

Analysis of SNV allele frequency differences between the P1 and P8A *L. interrogans* Lai strains revealed 25 intergenic SNVs, 22 on chromosome I, and three on chromosome II (Figure 1, Supplemental Table 1). In previous whole genome surveys, several ncRNA loci were detected in the *L. interrogans* Lai genome,^4^ including three cobalamin riboswitches that are expressed both in vivo and in vitro.^21^ Because these elements play vital roles in the regulation of gene expression, mutations within predicted ncRNAs could have functional implications potentially affecting virulence.

To evaluate whether any of our study identified intergenic SNVs resided in predicted ncRNA loci, we generated a list of predicted ncRNA in the *L. interrogans* Lai strain 56601 genome using RNAz^43^ and the nocoRNAc pipeline.^44^ Fifty candidate ncRNA loci were identified on chromosome I (cI replicon), and five on the cII replicon, none of which contained study-identified intergenic SNVs. Of the 55 candidate ncRNA loci identified, 41 were antisense to protein coding genes and 14 were found in intergenic regions (Table 2). Of these 14 ncRNA loci, none could be annotated using the Rfam database and could represent novel ncRNA genes.

## Discussion

This study analyzed genomic changes in a polyclonal population of *L. interrogans* serovar Lai strain 56601 that occur during the culture-based attenuation of a highly virulent parent strain into a nearly avirulent isogenic derivative. This analysis was carried out using a modified PLATYPUS pipeline, originally designed to analyze eukaryotic genomes, which was readily and accurately adapted for the analysis of *Leptospira* genomes (prokaryotic). Novel, potentially virulence-related genes were identified in this study by analyzing nsSNV allele frequency changes accompanying in vitro, culture attenuation of *L. interrogans* serovar Lai. Because of the stochasticity of the underlying processes giving rise to deleterious mutations in virulence-associated genes that are under neutral selection in vitro, future attenuation experiments would be most informative if whole genome sequencing data from several independent attenuated lineages are compared. The data summarized here will provide the foundation for future investigations to determine the role these genes play in the pathogenesis of leptospirosis.

Genome changes that occurred in the bacterial population during long-term in vitro culture passage attenuation of the virulent P1 *L. interrogans* Lai strain 56601 isolate into the avirulent P8A isolate likely occurred as the result of selection for rapid growth in vitro culture, likely to be in a tradeoff with virulence. After the isolation of the P1 strain from hamsters, the only selective pressure on the bacterial population became intrapopulation competition for growth in vitro in EMJH media. Therefore, the process of natural selection under these conditions would be expected to increase the population-level allelic frequencies of mutations beneficial to in vitro growth.^57^ Such changes are often accompanied by allele-frequency increases of mutations in genes necessary for growth in vivo (i.e., relaxed selection on virulence genes would lead to the accumulation of deleterious mutations in these genes during growth in vitro). Accordingly, nsSNVs otherwise deleterious to virulence in vivo, which had previously been kept at low frequencies by in vivo selection, for example, immune pressures of the host, would now be selectively neutral in vitro. These mutations would then be free to synchronously move with alleles under positive selection for growth in EMJH media in a type of genetic hitchhiking.^58^

Interestingly, all single nucleotide variations identified in our genomic analysis of the attenuated P8A isolate originated from existing low-frequency subpopulation alleles in the virulent P1 isolate. We did not find any spontaneous mutations arising during the in vitro attenuation process. Only preexisting mutations expanded in frequency based on statistically significant thresholds, a phenomenon also noted previously in the apicomplexan parasite *Babesia bovis*.^59^ The original process that generated these mutants appeared to have proceeded in a stochastic manner, SNVs appeared across the *L. interrogans* Lai genome with a nucleotide transition to transversion ratio, Ts/Tv, of approximately 0.5 (Supplemental Table 1), suggesting that at a given position a substitution of one nucleotide was just as likely as any other. All nsSNVs identified existed as minor variants to wild-type alleles in the P8A population (Figure 2A). Surprisingly, nsSNVs diverging from the reference sequence had an allele frequency of only 12% ± 4.97 (mean ± SD). It has been previously demonstrated in several other pathogens that microbial populations may harbor subpopulations that retain pathogenic capacity, despite being attenuated at the population level.^60^–^63^ Similarly, we were able to detect wild-type alleles in the majority of sequencing reads derived from the P8A isolate.

Two previous studies have examined genome differences between virulent and avirulent strains of *L. interrogans* serovar Lai to identify mutations that accompany the loss of the virulence of the parental strain. The first, which compared genome differences between *L. interrogans* serovar Lai strain IPAV (avirulent) and a non-isogenic isolate of *L. interrogans* serovar Lai,^64^ identified several hundred SNVs in gene-coding regions as well as dozens of insertions and deletions; interestingly many SNVs were found in genes related to signal transduction. The second study, recently reported from our group,^28^ identified a set of SNVs in 11 pathogen-specific genes of an attenuated isogenic derivative of *L. interrogans* serovar Lai strain 56601. There was no overlap in the genes identified in these earlier studies with those identified in this work, which mirrors results from another experimental evolution study in *Escherichia coli* that found few of the 115 strain replicates shared similar mutations.^57^ This lack of overlap strongly underscores the stochastic nature of SNV expansion in vitro*.* Nonetheless, it should be noted that the sequencing coverage was approximately 2.5X higher in this study relative to our previous study, reads were substantially longer here (100 bases versus 36) and paired ends were used. Also significant, this analysis detected mixed alleles, whereas our previous study focused only on dominant alleles. Because genes necessary for in vivo growth are under relaxed selection in vitro, the complement of putative virulence genes identified in attenuation experiments can differ substantially (i.e., no convergence), suggesting, that is, approach would be most informative if data derived from several independent attenuated lineages are analyzed.

Pathogenic *Leptospira* have evolved numerous signal transduction proteins to properly respond to environmental as well as in vivo host queues,^26^ in contrast to obligate parasites that have far fewer.^65^ Because pathogenic *Leptospira* are transmitted by soil and surface water, they must transition between the external and host environment.

The identification of nsSNVs in two GGDEF di-guanylate cyclase (DGC) signal transduction genes (LA_2704 and LA_2930, both previously shown to be upregulated during exposure to in vivo-like conditions; Figure 3) in our study was particularly intriguing. GGDEF domains catalyze the formation of the ubiquitous secondary messenger di-cyclic-GMP^66^ through a process of homo-dimerization of two DGC domains from separate proteins.^49^ Intracellular concentrations of di-c-GMP have been demonstrated experimentally to regulate several pathogenesis-related bacterial processes related to biofilm formation, motility, and virulence.^67^–^69^ The *L. interrogans* serovar Lai genome contains genes for 14 distinct GGDEF domain containing proteins,^26^ and members of the genus are known to produce biofilms both in vitro and in vivo.^70^,^71^ The physiological effects of di-c-GMP levels have been reviewed previously^47^; while intracellular di-c-GMP levels promote biofilm formation, they might have differential effects on (i.e., promote or inhibit) other phenotypes. While there are currently no experimental data regarding DGCs and di-c-GMP in *Leptospira*, it should be noted that di-c-GMP levels appear to positively regulate motility and virulence in other spirochetes.^72^–^74^

Data from a model biofilm system using *Pseudomonas aeruginosa* have demonstrated that increases in intracellular di-c-GMP levels, through the action of DGCs, lead to secretion of exopolysaccharide components required for biofilm formation.^75^ These polysaccharides then act as signals for DGCs in neighboring bacteria to increase their di-c-GMP levels, encouraging further exopolysaccharide secretion in a positive feedback mechanism similar to paracrine signaling in eukaryotes. Whether impaired GGDEF signaling causes a similar nonautonomous trait in *L. interrogans* remains undetermined, it is interesting to consider whether a small percentage of mutant cells (i.e., those harboring LA_2704 or LA_2930 nsSNV mutations) would influence the in vivo survival of the *Leptospira* population as a whole through impaired biofilm production.

Bacterial lipoproteins have been suggested to be involved in pathogenesis including adhesion to host cells, immune modulation, and the translocation of virulence factors into host cells,^76^,^77^ and there are several predicted in the genomes of spirochetes.^56^ Thus, the identification of mutations in the putative lipoprotein LA_3834 in this study is intriguing. While the function of this protein has yet to be determined experimentally, several independent lines of evidence point to LA_3834 being a part of the *Leptospira* virulence gene repertoire. In addition to being transcriptionally upregulated during in vivo surrogate experiments,^18^,^20^,^21^ LA_3834 was recently demonstrated to be under the control of a transcriptional regulator (LB_139) that when knocked out, decreased expression of several genes (including LA_3834) and attenuated virulence in a hamster model of leptospirosis.^22^

Two other study-identified genes (*LA_2950* and *LA_3455*) with nsSNVs at conserved residues may also be important to *Leptospira* virulence and survival in vivo. LA_2950 encodes a protein predicted PDZ serine protease. Studies in *Salmonella typhimurium* have demonstrated that other PDZ serine proteases participate in the in vivo stress response to host microbicidal pressures.^78^–^80^ Bacteria with mutations in these genes were attenuated compared with wild-type parental strains, with decreased tissue burdens (up to a 10^5^-fold decrease in one study).^80^ LA_3455 encodes the *Leptospira* GlpF glycerol uptake facilitator protein. *L. interrogans* cannot use sugars as carbon sources, but instead, synthesizes sugars with de novo gluconeogenesis from glycerol.^27^ Since the nonsynonymous S56P SNV identified in the P8 strain of our study may introduce a strain in the secondary structure of a conserved helix essential for function, *Leptospira* cells harboring this mutation could conceivably experience an impaired acquisition of glycerol in vivo that could have downstream biosynthetic consequences.

To the best of our knowledge, our generation of a list of computationally predicted ncRNAs in *L. interrogans* is the first in the field. Although none of our study-identified intergenic SNVs mapped to these regions, small noncoding RNAs have recently been shown to regulate pathogenic mechanisms in bacteria.^81^–^84^ Thus, it would be important to test whether similar mechanisms exist in pathogenic *Leptospira.*

This study has limitations, primarily in that further work needs to be done both qualitatively and quantitatively to describe the individual contribution of the genes identified here to *Leptospira* pathogenesis. It is likely that the genes identified in this study may be part of a virulence-related transcriptional profile, and the increase in nsSNV alleles seen may collectively reduce the pathogenicity of *Leptospira* with these mutations. The ultimate mechanism of attenuation of *L. interrogans* serovar Lai in this study appears to be the additive effect of multiple mutant alleles, each subtracting from overall population fitness in vivo. The individual contributions of each of these genes to overall virulence is likely to remain hazy until more reliable methods of targeted mutagenesis are established for this important pathogen, until then attenuation-based studies are a reasonable alternative for identifying putative virulence-associated genes.

## Supplementary Material

Supplemental Table.

## Figures and Tables

**Table 1:** Genome alignment statistics for *Leptospira interrogans* serovar Lai strains P1 and P8A

Sample	Median insert size (bp)	Total reads	Aligned reads (%)	Mean coverage	% Bases > 20X	% Bases > 40X	% Bases > 100X	% Bases > 130X	% Bases > 150X
P1	113	15,583,466	15,492,436 (99.4)	250.49	100	99.9	98.8	94.9	89.6
P8	129	15,668,032	15,651,273 (99.9)	263.53	100	99.9	98.4	95.5	91.8

**Table 2:** Predicted ncRNAs in *Leptospira interrogans* serovar Lai

Chromosome	Start (bp)	Stop (bp)	Strand	SIDD value	Terminator confidence
CI	235,751	235,834	−	−0.5	73
CI	422,027	422,128	−	3.5	100
CI	538,202	538,259	+	2.5	72
CI	548,190	548,249	−	3.2	100
CI	1,181,513	1,181,570	+	2.0	74
CI	2,172,984	2,173,177	−	−0.2	76
CI	2,410,576	2,410,725	−	−0.3	78
CI	2,545,112	2,545,233	−	2.9	77
CI	2,823,520	2,823,576	−	−0.5	100
CI	2,876,638	2,876,810	−	−0.5	80
CI	2,935,474	2,935,522	−	−1.0	70
CI	3,382,029	3,382,080	+	2.2	89
CI	4,152,345	4,152,404	+	2.6	79
CII	73,532	73,627	−	2.5	86

In silico predicted ncRNAs in the *L. interrogans* serovar Lai genome. The 14 predicted ncRNAs are listed by chromosome, start/stop positions, as well as the DNA strand the site resides on. Stress-induced duplex destabilization (SIDD) values near zero are highly destabilized states that promote helicase action and transcription. Terminator confidence is a percentage of certainty of transcription stop sites.
