# Supplementary material for: Whole Genome Shotgun Sequencing Shows Selection on Leptospira Regulatory Proteins during in vitro Culture Attenuation
Source: Am J Trop Med Hyg. 2016 Feb 3;94(2):302–13. doi: 10.4269/ajtmh.15-0401 (PMC4751964; doi:10.4269/ajtmh.15-0401)
Supplement: Supplementary file 1 [file SD3.pdf]

Supplemental Table 1

Genome coordinates of SNVs with a change in allelic frequency

Chrom	Position	Ref	ALT	z score	P value	Bonferroni_ corr_P value	Filter	No of total PI reads	No of PI ALT reads	No of total Ps reads	No of PS ALT reads	Abs value diff of proportion	Mutation type	Mutation severity	Mutation result	Codon change	AA change	Gene	Gene description
LiLai_01	68,355	C	T	2.43,947	1.47E-02	1.00E+00	Pass	DP1 = 372	N1 = 63	DP2 = 412	N2 = 45	0.0,601,315	Nonsynonymous coding	Moderate	Missense	Gaa/Aaa	E86K	cysE	Transcript_LA_0062
LiLai_01	94,757	G	A	3.55,639	3.76E-04	3.72E-02	Pass	DP1 = 484	N1 = 21	DP2 = 497	N2 = 51	0.0,592,273	Intergenic	Modifier					
LiLai_01	94,766	G	T	3.19,858	1.38E-03	1.37E-01	Pass	DP1 = 554	N1 = 29	DP2 = 580	N2 = 60	0.0,511,017	Intergenic	Modifier					
LiLai_01	94,767	T	A	3.19,858	1.38E-03	1.37E-01	Pass	DP1 = 554	N1 = 29	DP2 = 580	N2 = 60	0.0,511,017	Intergenic	Modifier					
LiLai_01	94,771	T	G	3.23,081	1.23E-03	1.22E-01	Pass	DP1 = 544	N1 = 32	DP2 = 574	N2 = 65	0.0,544,169	Intergenic	Modifier					
LiLai_01	94,828	A	G	3.22,902	1.24E-03	1.23E-01	Pass	DP1 = 614	N1 = 116	DP2 = 701	N2 = 185	0.0,749,836	Intergenic	Modifier					
LiLai_01	265,328	C	T	2.08,198	3.73E-02	1.00E+00	Pass	DP1 = 126	N1 = 54	DP2 = 144	N2 = 80	0.126,984	Nonsynonymous coding	Moderate	Missense	Gga/Aga	G176R	LA_0269	Transcript_LA_0269
LiLai_01	269,171	G	T	2.05,326	4.00E-02	1.00E+00	Pass	DP1 = 246	N1 = 29	DP2 = 260	N2 = 17	0.0,525,016	Nonsynonymous coding	Moderate	Missense	Gat/Tat	D178Y	lolE	Transcript_LA_0273
LiLai_01	276,615	C	T	2.13,095	3.31E-02	1.00E+00	Pass	DP1 = 114	N1 = 1	DP2 = 98	N2 = 6	0.0,524,526	Intergenic	Modifier					
LiLai_01	516,271	T	A	2.57,247	1.01E-02	1.00E+00	Pass	DP1 = 189	N1 = 16	DP2 = 357	N2 = 12	0.0,510,426	Intergenic	Modifier					
LiLai_01	656,535	G	A	2.16,161	3.06E-02	1.00E+00	Pass	DP1 = 232	N1 = 20	DP2 = 217	N2 = 33	0.0,658,668	Intergenic	Modifier					
LiLai_01	697,473	A	C	2.31,806	2.04E-02	1.00E+00	Pass	DP1 = 170	N1 = 19	DP2 = 239	N2 = 12	0.0,615,555	Nonsynonymous coding	Moderate	Missense	gTt/gGt	V39G	lysC	Transcript_LA_0693
LiLai_01	721,535	A	G	2.63,288	8.47E-03	8.38E-01	Pass	DP1 = 325	N1 = 12	DP2 = 297	N2 = 26	0.050,619	Nonsynonymous coding	Low	Silent	aaT/aaC	N6	LA_0707	Transcript_LA_0707
LiLai_01	784,762	G	C	3.05,086	2.28E-03	2.26E-01	Pass	DP1 = 203	N1 = 1	DP2 = 246	N2 = 14	0.0,519,845	Nonsynonymous coding	Low	Silent	gtG/gtC	V228	LA_0775	Transcript_LA_0775
LiLai_01	787,340	G	T	2.34,244	1.92E-02	1.00E+00	Pass	DP1 = 134	N1 = 19	DP2 = 137	N2 = 35	0.113,683	Nonsynonymous coding	Moderate	Missense	aCc/aAc	T145N	LA_0776	Transcript_LA_0776
LiLai_01	990,824	C	A	2.44,907	1.43E-02	1.00E+00	Pass	DP1 = 115	N1 = 8	DP2 = 156	N2 = 2	0.0,567,447	Stop gained	High	Missense	Gaa/Taa	E44*	cdc9	Transcript_LA_0984
LiLai_01	1,210,481	A	T	2.22,242	2.63E-02	1.00E+00	Pass	DP1 = 115	N1 = 15	DP2 = 137	N2 = 7	0.0,793,399	Nonsynonymous coding	Moderate	Missense	tAt/tTt	Y139F	LA_1209	Transcript_LA_1209
LiLai_01	1,210,488	T	A	2.16,266	3.06E-02	1.00E+00	Pass	DP1 = 103	N1 = 17	DP2 = 113	N2 = 8	0.0942521	Nonsynonymous coding	Moderate	Missense	aaT/aaA	N141K	LA_1209	Transcript_LA_1209
LiLai_01	1,210,499	A	T	2.24,494	2.48E-02	1.00E+00	Pass	DP1 = 92	N1 = 7	DP2 = 97	N2 = 1	0.0,657,777	Nonsynonymous coding	Moderate	Missense	tAt/tTt	Y145F	LA_1209	Transcript_LA_1209
LiLai_01	1,210,502	A	T	1.99,443	4.61E-02	1.00E+00	Pass	DP1 = 95	N1 = 6	DP2 = 100	N2 = 1	0.0,531,579	Nonsynonymous coding	Moderate	Missense	tAt/tTt	Y146F	LA_1209	Transcript_LA_1209
LiLai_01	1,242,872	A	G	2.70,342	6.86E-03	6.79E-01	Pass	DP1 = 133	N1 = 0	DP2 = 245	N2 = 13	0.0,530,612	Nonsynonymous coding	Low	SILENT	ggA/ggG	G104	thrS	Transcript_LA_1240
LiLai_01	1,387,201	T	G	2.20,793	2.72E-02	1.00E+00	Pass	DP1 = 144	N1 = 3	DP2 = 187	N2 = 14	0.054,033	Nonsynonymous coding	Moderate	Missense	gTt/gGt	V283G	rbsK	Transcript_LA_1392
LiLai_01	1,387,207	A	T	2.40,704	1.61E-02	1.00E+00	Pass	DP1 = 169	N1 = 8	DP2 = 195	N2 = 23	0.0,706,114	Nonsynonymous coding	Moderate	Missense	tAt/tTt	Y285F	rbsK	Transcript_LA_1392
LiLai_01	1,401,957	A	C	3.41,812	6.31E-04	6.24E-02	Pass	DP1 = 202	N1 = 2	DP2 = 171	N2 = 14	0.0,719,704	Intergenic	Modifier					
LiLai_01	1,460,743	A	T	2.54,199	1.10E-02	1.00E+00	Pass	DP1 = 226	N1 = 6	DP2 = 337	N2 = 26	0.0,506,027	Intergenic	Modifier					
LiLai_01	1,460,748	T	G	2.57,097	1.01E-02	1.00E+00	Pass	DP1 = 215	N1 = 6	DP2 = 331	N2 = 27	0.053,664	Intergenic	Modifier					
LiLai_01	1,733,977	T	G	2.00,227	4.53E-02	1.00E+00	Pass	DP1 = 204	N1 = 28	DP2 = 238	N2 = 50	0.0,728,291	Nonsynonymous coding	Moderate	Missense	gTt/gGt	V130G	cheB	Transcript_LA_1744
LiLai_01	1,751,891	T	C	1.98,431	4.72E-02	1.00E+00	Pass	DP1 = 1	N1 = 1	DP2 = 8	N2 = 1	0.875	Nonsynonymous coding	Moderate	Missense	aTc/aCc	I113T	LA_1761	Transcript_LA_1761
LiLai_01	1,793,250	G	T	2.53,798	1.11E-02	1.00E+00	Pass	DP1 = 220	N1 = 55	DP2 = 272	N2 = 43	0.0,919,118	Nonsynonymous coding	Moderate	Missense	aCt/aAt	T36N	LA_1812	Transcript_LA_1812
LiLai_01	1,934,172	T	G	2.18,146	2.91E-02	1.00E+00	Pass	DP1 = 260	N1 = 40	DP2 = 318	N2 = 30	0.0,595,065	Nonsynonymous coding	Moderate	Missense	Tcc/Gcc	S32A	LA_1966	Transcript_LA_1966
LiLai_01	2,161,018	T	G	2.15,648	3.10E-02	1.00E+00	Pass	DP1 = 304	N1 = 43	DP2 = 283	N2 = 24	0.0,566,417	Nonsynonymous coding	Moderate	Missense	gTa/gGa	V1135G	LA_2186	Transcript_LA_2186

(continued)

Supplemental Table 1
Continued

Chrom	Position	Ref	ALT	z score	P value	Bonferroni_corr_P value	Filter	No of total PI reads	No of PI ALT reads	No of total Ps reads	No of PS ALT reads	Abs value diff of proportion	Mutation type	Mutation severity	Mutation result	Codon change	AA change	Gene	Gene description
LiLai_01	2,182,705	A	C	2.53,207	1.13E-02	1.00E+00	Pass	DP1 = 140	N1 = 15	DP2 = 116	N2 = 3	0.0,812,808	Nonsynonymous coding	Moderate	Missense	Ttt/Gtt	F46V	LA_2204	Transcript_LA_2204
LiLai_01	2,202,482	A	T	2.30,147	2.14E-02	1.00E+00	Pass	DP1 = 219	N1 = 17	DP2 = 278	N2 = 40	0.0,662,593	Nonsynonymous coding	Moderate	Missense	Atg/Ttg	M435L	mgtA	Transcript_LA_2221
LiLai_01	2,202,499	A	T	2.29,593	2.17E-02	1.00E+00	Pass	DP1 = 211	N1 = 23	DP2 = 265	N2 = 49	0.0,759,009	Nonsynonymous coding	Moderate	Missense	ttA/ttT	L440F	mgtA	Transcript_LA_2221
LiLai_01	2,202,538	G	T	3.13,719	1.71E-03	1.69E-01	Pass	DP1 = 236	N1 = 13	DP2 = 266	N2 = 37	0.08,4013	Nonsynonymous coding	Moderate	Missense	ttG/ttT	L453F	mgtA	Transcript_LA_2221
LiLai_01	2,341,072	C	T	1.9,615	4.98E-02	1.00E+00	Pass	DP1 = 247	N1 = 60	DP2 = 240	N2 = 41	0.0,720,816	Nonsynonymous coding	Moderate	Missense	Gaa/Aaa	E4K	LA_2365	Transcript_LA_2365
LiLai_01	2,359,781	A	G	1.96,007	5.00E-02	1.00E+00	Pass	DP1 = 262	N1 = 36	DP2 = 283	N2 = 24	0.0,525,989	Nonsynonymous coding	Low	Silent	ccT/ccC	P33	rnhB	Transcript_LA_2386
LiLai_01	2,493,859	A	G	2.51,778	1.18E-02	1.00E+00	Pass	DP1 = 164	N1 = 5	DP2 = 219	N2 = 21	0.0,654,026	Nonsynonymous coding	Low	Silent	ggT/ggC	G206	hisD	Transcript_LA_2515
LiLai_01	2,502,662	A	T	2.26,928	2.33E-02	1.00E+00	Pass	DP1 = 309	N1 = 33	DP2 = 273	N2 = 15	0.0,518,511	Intergenic	Modifier					
LiLai_01	2,685,141	G	T	2.13,319	3.29E-02	1.00E+00	Pass	DP1 = 186	N1 = 21	DP2 = 256	N2 = 48	0.0,745,968	Nonsynonymous coding	Moderate	Missense	tGt/tTt	C311F	LA_2704	Transcript_LA_2704
LiLai_01	2,821,742	C	A	2.43,475	1.49E-02	1.00E+00	Pass	DP1 = 222	N1 = 7	DP2 = 274	N2 = 23	0.0,524,101	Nonsynonymous coding	Moderate	Missense	Cac/Aac	H108N	argF	Transcript_LA_2840
LiLai_01	2,828,198	T	C	2.45,221	1.42E-02	1.00E+00	Pass	DP1 = 167	N1 = 11	DP2 = 221	N2 = 32	0.0,789,281	Nonsynonymous coding	Low	Silent	ggA/ggG	G319	LA_2847	Transcript_LA_2847
LiLai_01	2,879,015	T	A	2.33,077	1.98E-02	1.00E+00	Pass	DP1 = 202	N1 = 5	DP2 = 154	N2 = 12	0.0,531,696	Intergenic	Modifier					
LiLai_01	2,903,205	A	T	3.22,773	1.25E-03	1.24E-01	Pass	DP1 = 162	N1 = 6	DP2 = 183	N2 = 25	0.099,575	Nonsynonymous coding	Moderate	Missense	tAt/tTt	Y94F	LA_2930	Transcript_LA_2930
LiLai_01	2,921,917	C	G	2.21,928	2.65E-02	1.00E+00	Pass	DP1 = 140	N1 = 3	DP2 = 124	N2 = 10	0.0,592,166	Nonsynonymous coding	Moderate	Missense	cCg/cGg	P81R	LA_2950	Transcript_LA_2950
LiLai_01	3,003,110	T	C	2.16,945	3.00E-02	1.00E+00	Pass	DP1 = 331	N1 = 41	DP2 = 260	N2 = 49	0.0,645,945	Nonsynonymous coding	Low	Silent	ggA/ggG	G408	LA_3029	Transcript_LA_3029
LiLai_01	3,023,838	C	T	4.85,973	1.18E-06	1.16E-04	Pass	DP1 = 302	N1 = 163	DP2 = 356	N2 = 125	0.188,612	Nonsynonymous coding	Moderate	Missense	Gca/Aca	A101T	LA_3060	Transcript_LA_3060
LiLai_01	3,028,216	T	A	2.4,346	1.49E-02	1.00E+00	Pass	DP1 = 160	N1 = 2	DP2 = 153	N2 = 10	0.0,528,595	Nonsynonymous coding	Moderate	Missense	ttA/ttT	L2465F	LA_3067	Transcript_LA_3067
LiLai_01	3,183,571	A	C	2.83,412	4.60E-03	4.55E-01	Pass	DP1 = 228	N1 = 30	DP2 = 528	N2 = 36	0.0,633,971	Intergenic	Modifier					
LiLai_01	3,183,595	T	C	2.68,743	7.20E-03	7.13E-01	Pass	DP1 = 221	N1 = 27	DP2 = 548	N2 = 35	0.0,583,033	Intergenic	Modifier					
LiLai_01	3,191,254	T	C	2.0,969	3.60E-02	1.00E+00	Pass	DP1 = 121	N1 = 14	DP2 = 172	N2 = 36	0.0,935,998	Nonsynonymous coding	Moderate	Missense	Atg/Gtg	M856V	LA_3207	Transcript_LA_3207
LiLai_01	3,205,037	T	A	2.97,949	2.89E-03	2.86E-01	Pass	DP1 = 153	N1 = 0	DP2 = 88	N2 = 5	0.0,568,182	Intergenic	Modifier					
LiLai_01	3,205,090	G	A	2.58,191	9.83E-03	9.73E-01	Pass	DP1 = 174	N1 = 6	DP2 = 107	N2 = 12	0.0,776,668	Intergenic	Modifier					
LiLai_01	3,205,093	G	A	2.37,724	1.74E-02	1.00E+00	Pass	DP1 = 185	N1 = 8	DP2 = 122	N2 = 14	0.0,715,109	Intergenic	Modifier					
LiLai_01	3,205,102	G	A	2.32,907	1.99E-02	1.00E+00	Pass	DP1 = 195	N1 = 15	DP2 = 132	N2 = 21	0.0,821,678	Intergenic	Modifier					
LiLai_01	3,226,045	C	T	3.55,903	3.72E-04	3.69E-02	Pass	DP1 = 946	N1 = 205	DP2 = 976	N2 = 150	0.0,630,134	Synonymous coding	Low	Silent	atC/atT	I77	LA_3249	Transcript_LA_3249
LiLai_01	3,226,573	A	G	2.04,565	4.08E-02	1.00E+00	Pass	DP1 = 481	N1 = 96	DP2 = 578	N2 = 146	0.053,011	Nonsynonymous coding	Moderate	Missense	Aat/Gat	N3D	LA_3250	Transcript_LA_3250
LiLai_01	3,240,929	T	C	3.46,549	5.29E-04	5.24E-02	Pass	DP1 = 148	N1 = 4	DP2 = 194	N2 = 26	0.106,994	Synonymous coding	Low	Silent	ggA/ggG	G694	LA_3267	Transcript_LA_3267
LiLai_01	3,242,009	T	C	2.23,016	2.57E-02	1.00E+00	Pass	DP1 = 132	N1 = 12	DP2 = 186	N2 = 6	0.058,651	Synonymous coding	Low	Silent	ggA/ggG	G334	LA_3267	Transcript_LA_3267
LiLai_01	3,268,692	A	G	2.3,257	2.00E-02	1.00E+00	Pass	DP1 = 176	N1 = 10	DP2 = 214	N2 = 27	0.06,935	Synonymous coding	Low	Silent	ccT/ccC	P320	dxr	Transcript_LA_3292

(continued)

Supplemental Table 1
Continued

Chrom	Position	Ref	ALT	z score	P value	Bonferroni_corr_P value	Filter	No of total PI reads	No of PI ALT reads	No of total Ps reads	No of PS ALT reads	Abs value diff of proportion	Mutation type	Mutation severity	Mutation result	Codon change	AA change	Gene	Gene description
LiLai_01	3,275,393	G	T	2.26,535	2.35E-02	1.00E+00	Pass	DP1 = 148	N1 = 4	DP2 = 224	N2 = 19	0.0,577,944	Nonsynonymous coding	Moderate	Missense	aCg/aAg	T338K	LA_3303	Transcript_LA_3303
LiLai_01	3,275,404	T	C	2.09,523	3.62E-02	1.00E+00	Pass	DP1 = 151	N1 = 16	DP2 = 215	N2 = 40	0.0,800,862	Synonymous coding	Low	Silent	ggA/ggG	G334	LA_3303	Transcript_LA_3303
LiLai_01	3,358,827	A	C	2.21,453	2.68E-02	1.00E+00	Pass	DP1 = 190	N1 = 29	DP2 = 238	N2 = 20	0.068,598	Nonsynonymous coding	Moderate	Missense	gTt/gGt	V92G	LA_3390	Transcript_LA_3390
LiLai_01	3,370,773	T	G	1.96,298	4.96E-02	1.00E+00	Pass	DP1 = 162	N1 = 7	DP2 = 252	N2 = 24	0.0,520,282	Nonsynonymous coding	Moderate	Missense	gTt/gGt	V102G	LA_3402	Transcript_LA_3402
LiLai_01	3,376,590	T	A	3.21,513	1.30E-03	1.29E-01	Pass	DP1 = 302	N1 = 6	DP2 = 364	N2 = 27	0.0,543,083	Nonsynonymous coding	Moderate	Missense	Aca/Tca	T53S	alaS	Transcript_LA_3407
LiLai_01	3,414,780	T	A	2.20,029	2.78E-02	1.00E+00	Pass	DP1 = 148	N1 = 6	DP2 = 170	N2 = 18	0.0,653,418	Intergenic	Modifier					
LiLai_01	3,414,782	C	T	2.8,892	3.86E-03	3.82E-01	Pass	DP1 = 149	N1 = 38	DP2 = 171	N2 = 22	0.126,379	Intergenic	Modifier					
LiLai_01	3,414,806	C	T	2.10,619	3.52E-02	1.00E+00	Pass	DP1 = 197	N1 = 11	DP2 = 200	N2 = 23	0.0,591,624	Nonsynonymous coding	Moderate	Missense	Ctt/Ttt	L5F	LA_3450	Transcript_LA_3450
LiLai_01	3,422,655	T	C	2.0,868	3.69E-02	1.00E+00	Pass	DP1 = 151	N1 = 8	DP2 = 224	N2 = 26	0.0,630,913	Nonsynonymous coding	Moderate	Missense	Tcc/Ccc	S56P	glpF	Transcript_LA_3455
LiLai_01	3,429,364	T	A	2.62,308	8.71E-03	8.63E-01	Pass	DP1 = 153	N1 = 18	DP2 = 180	N2 = 41	0.110,131	Nonsynonymous coding	Moderate	Missense	aaT/aaA	N127K	glcD	Transcript_LA_3461
LiLai_01	3,429,374	G	A	2.51,097	1.20E-02	1.00E+00	Pass	DP1 = 174	N1 = 18	DP2 = 203	N2 = 40	0.0,935,961	Nonsynonymous coding	Moderate	Missense	Gaa/Aaa	E131K	glcD	Transcript_LA_3461
LiLai_01	3,429,394	C	T	2.61,224	9.00E-03	8.91E-01	Pass	DP1 = 181	N1 = 6	DP2 = 209	N2 = 21	0.0,673,293	Synonymous coding	Low	Silent	ttC/ttT	F137	glcD	Transcript_LA_3461
LiLai_01	3,454,220	T	C	2.76,385	5.71E-03	5.66E-01	Pass	DP1 = 3	N1 = 1	DP2 = 22	N2 = 0	0.333,333	Intergenic	Modifier					
LiLai_01	3,552,182	C	T	2.61,784	8.85E-03	8.76E-01	Pass	DP1 = 131	N1 = 10	DP2 = 151	N2 = 2	0.0,630,908	Nonsynonymous coding	Moderate	Missense	tCt/tTt	S39F	wecE	Transcript_LA_3597
LiLai_01	3,667,225	A	G	2.6,223	8.73E-03	8.65E-01	Pass	DP1 = 141	N1 = 9	DP2 = 223	N2 = 3	0.0,503,769	Synonymous coding	Low	Silent	ggA/ggG	G115	dnaJ	Transcript_LA_3706
LiLai_01	3,667,411	C	G	2.1,496	3.16E-02	1.00E+00	Pass	DP1 = 142	N1 = 17	DP2 = 214	N2 = 12	0.0,636,435	Synonymous coding	Low	Silent	ggC/ggG	G177	dnaJ	Transcript_LA_3706
LiLai_01	3,667,464	G	T	2.61,743	8.86E-03	8.77E-01	Pass	DP1 = 137	N1 = 9	DP2 = 216	N2 = 3	0.0,518,045	Nonsynonymous coding	Moderate	Missense	tGc/tTc	C195F	dnaJ	Transcript_LA_3706
LiLai_01	3,689,219	A	C	2.21,154	2.70E-02	1.00E+00	Pass	DP1 = 191	N1 = 15	DP2 = 252	N2 = 37	0.0,682,914	Nonsynonymous coding	Moderate	Missense	Tta/Gta	L1624V	LA_3725	Transcript_LA_3725
LiLai_01	3,726,955	A	G	2.6,455	8.16E-03	8.08E-01	Pass	DP1 = 138	N1 = 2	DP2 = 105	N2 = 9	0.0,712,215	Synonymous coding	Low	Silent	ggA/ggG	G444	LA_3751	Transcript_LA_3751
LiLai_01	3,729,570	A	G	1.98,836	4.68E-02	1.00E+00	Pass	DP1 = 54	N1 = 0	DP2 = 28	N2 = 2	0.0,714,286	Intergenic	Moderate					
LiLai_01	3,745,583	G	A	2.24,236	2.49E-02	1.00E+00	Pass	DP1 = 174	N1 = 14	DP2 = 160	N2 = 4	0.0,554,598	Synonymous coding	Low	Silent	gaG/gaA	E67	LA_3774	Transcript_LA_3774
LiLai_01	3,776,977	A	C	2.26,571	2.35E-02	1.00E+00	Pass	DP1 = 303	N1 = 67	DP2 = 372	N2 = 57	0.0,678,963	Synonymous coding	Low	Silent	acA/acC	T125	amtB	Transcript_LA_3806
LiLai_01	3,804,775	C	T	2.57,917	9.90E-03	9.80E-01	Pass	DP1 = 254	N1 = 7	DP2 = 168	N2 = 14	0.0,557,743	Nonsynonymous coding	Moderate	Missense	Ctt/Ttt	L8F	LA_3834	Transcript_LA_3834
LiLai_01	3,867,444	G	T	2.76,748	5.65E-03	5.59E-01	Pass	DP1 = 220	N1 = 17	DP2 = 207	N2 = 4	0.0,579,491	Nonsynonymous coding	Moderate	Missense	Caa/Aaa	Q229K	LA_3894	Transcript_LA_3894
LiLai_01	4,062,966	A	C	2.2,934	2.18E-02	1.00E+00	Pass	DP1 = 304	N1 = 26	DP2 = 296	N2 = 43	0.059,744	Nonsynonymous coding	Moderate	Missense	gTc/gGc	V189G	adhP	Transcript_LA_4085
LiLai_01	4,134,583	T	G	2.35,767	1.84E-02	1.00E+00	Pass	DP1 = 202	N1 = 18	DP2 = 249	N2 = 9	0.0,529,643	Nonsynonymous coding	Moderate	Missense	agT/agG	S73R	LA_4159	Transcript_LA_4159
LiLai_01	433,1254	G	T	2.98,301	2.85E-03	2.83E-01	Pass	DP1 = 258	N1 = 42	DP2 = 234	N2 = 64	0.110,714	Nonsynonymous coding	Moderate	Missense	aCt/aAt	T36N	LA_4353	Transcript_LA_4353

(continued)

Supplemental Table 1
Continued

Chrom	Position	Ref	ALT	z score	P value	Bonferroni_ corr_P value	Filter	No of total PI reads	No of PI ALT reads	No of total Ps reads	No of PS ALT reads	Abs value diff of proportion	Mutation type	Mutation severity	Mutation result	Codon change	AA change	Gene	Gene description
LiLai_02	38,522	A	G	2.66,976	7.59E-03	7.51E-01	Pass	DP1 = 211	N1 = 51	DP2 = 244	N2 = 35	0.0,982,635	Nonsynonymous coding	Moderate	Missense	Ata/Gta	I235V	LB_048	Transcript_LB_048
LiLai_02	38,528	A	G	2.48,924	1.28E-02	1.00E++00	Pass	DP1 = 214	N1 = 51	DP2 = 239	N2 = 35	0.0,918,742	Nonsynonymous coding	Moderate	Missense	Aaa/Gaa	K237E	LB_048	Transcript_LB_048
LiLai_02	79,520	T	G	2.37,896	1.74E-02	1.00E+00	Pass	DP1 = 408	N1 = 52	DP2 = 352	N2 = 67	0.0,628,899	Nonsynonymous coding	Low	Silent	ggT/ggG	G114	LB_089	Transcript_LB_089
LiLai_02	104,164	C	G	4.50,129	6.75E-06	6.69E-04	Pass	DP1 = 279	N1 = 1	DP2 = 289	N2 = 23	0.0,760,005	Nonsynonymous coding	Moderate	Missense	aCg/aGg	T381R	LB_111	Transcript_LB_111
LiLai_02	104,196	T	G	3.08,812	2.01E-03	1.99E-01	Pass	DP1 = 342	N1 = 23	DP2 = 295	N2 = 5	0.0,503,023	Nonsynonymous coding	Moderate	Missense	Ttt/Gtt	F392V	LB_111	Transcript_LB_111
LiLai_02	152,966	G	A	2.2,261	2.60E-02	1.00E+00	Pass	DP1 = 279	N1 = 45	DP2 = 250	N2 = 24	0.0,652,903	Nonsynonymous coding	Moderate	Missense	Ctt/Ttt	L166F	cbiH	Transcript_LB_157
LiLai_02	226,487	A	G	4.69,207	2.70E-06	2.68E-04	Pass	DP1 = 278	N1 = 43	DP2 = 424	N2 = 132	0.156,644	Nonsynonymous coding	Low	Silent	gaA/gaG	E67	LB_230	Transcript_LB_230
LiLai_02	271,358	A	T	2.6,173	8.86E-03	8.77E-01	Pass	DP1 = 290	N1 = 10	DP2 = 316	N2 = 27	0.0,509,603	Nonsynonymous coding	Moderate	Missense	tAt/tTt	Y380F	mcm2	Transcript_LB_273
LiLai_02	271,400	A	G	1.97,076	4.88E-02	1.00E+00	Pass	DP1 = 264	N1 = 44	DP2 = 277	N2 = 65	0.0679,904	Nonsynonymous coding	Moderate	Missense	gAa/gGa	E394G	mcm2	Transcript_LB_273
LiLai_02	302,735	T	C	3.31,573	9.14E-04	9.05E-02	Pass	DP1 = 312	N1 = 73	DP2 = 411	N2 = 143	0.113,958	Synonymous coding	Low	Silent	gaA/gaG	E67	LB_304	Transcript_LB_304
LiLai_02	305,553	T	A	2.5,467	1.09E-02	1.00E++00	Pass	DP1 = 246	N1 = 20	DP2 = 198	N2 = 5	0.0560,483	Intergenic	Modifier					
LiLai_02	318,569	A	G	2.81,945	4.81E-03	4.76E-01	Pass	DP1 = 226	N1 = 5	DP2 = 307	N2 = 24	0.056,052	Nonsynonymous coding	Moderate	Missense	Tca/Cca	S177P	LB_322	Transcript_LB_322

ALT = alternate; AA = amino acid; abs = absolute.
